# Effects of age and period on functional dentition: an analysis of skin color/race inequalities in the 2003–2023 National Oral Health Surveys (SB Brasil)

**DOI:** 10.1590/1980-549720260033.supl.1

**Published:** 2026-10-19

**Authors:** Maria Helena Rodrigues Galvão, Sophia Queiroz Marques dos Santos, Niely Enetice de Sousa Catão, Ane Polline Lacerda Protasio

**Affiliations:** I Universidade Federal de Pernambuco – Vitória de Santo Antão (PE), Brazil.; II Universidade Federal do Rio Grande do Norte – Natal (RN), Brazil.; III Universidade Federal da Paraíba – João Pessoa (PB), Brazil.

**Keywords:** Health surveys, Oral health, Tooth loss, Secondary data analysis, Life cycle

## Abstract

**Objective:**

To estimate the interaction between age and period in the maintenance of functional dentition among Brazilian adults and older adults in 2003, 2010, and 2023 and to investigate inequalities by skin color or race.

**Methods:**

Data from the 2003, 2010, and 2023 National Oral Health Surveys were analyzed, forming a time series of repeated cross-sectional studies with 54,557 participants aged 35–44 and 65–74 years. Prevalence ratios (PR) were estimated using quasi-Poisson regression, including terms for age, period, and age-period interaction, with and without adjustment for skin color or race, considering the complex sample design.

**Results:**

The age-period interaction was significant (p<0.001), indicating that temporal changes differed between age groups. Functional dentition prevalence increased from 52.5% (95%CI 48.8–56.1) in 2003 to 73.6% (95%CI 71.4–75.8) in 2023. Among adults, it increased from 71.5 to 96.1%, while among older adults there was a reduction between 2003 and 2010, but in 2023 it was more than twice that observed in 2003. Interaction terms for 2023 were positive and increased with age. After adjusting for skin color or race, mixed-race individuals (PR 0.91; 95%CI 0.88–0.94) and Black individuals (PR 0.95; 95%CI 0.91–0.99) presented lower functional dentition prevalence compared to white individuals, with no significant differences for the remaining groups.

**Conclusions:**

Functional dentition maintenance showed a significant increase between 2003 and 2023, but with distinct trajectories between adults and older adults, highlighting delayed advances in the older adult population. Inequalities by skin color or race persisted, mainly affecting Black and mixed-race individuals.

## INTRODUCTION

Tooth loss is one of the most serious outcomes in oral health and reflects accumulated exposure to social, economic, behavioral conditions, and access to health services throughout life^1,2^. Among its indicators, functional dentition, which can be defined by the presence of at least 20 natural teeth^3^, represents an outcome of greater relevance to public health because it preserves masticatory, phonetic, aesthetic, and social functions, being associated with quality of life and healthy aging^1,2^.

According to the National Oral Health Surveys (SB Brasil), there has been a progressive improvement in oral health conditions of the Brazilian population in recent decades, evidenced by the reduction of edentulism and the increase in dental preservation. These changes occurred in a context of social transformations and expansion of public oral health policies; however, their benefits may not have been distributed in a homogeneous way between different population segments^4-6^.

In Brazil, studies on tooth loss and functional dentition have been predominantly population-based and cross-sectional surveys. There is evidence of the persistence of inequalities according to skin color or race, income and level of education, with lower prevalence of functional dentition among Black people and socially disadvantaged groups, despite the improvement observed in population indicators^4,5,7^. Nonetheless, whether this evolution has occurred in a similar way between adults and older adults, and whether the effects of changes observed over time on functional dentition have differed between age groups remain little investigated.

This issue is particularly relevant in the light of the epidemiology of the course of life, according to which health in adulthood and old age results from the accumulation of biological, social, and material exposures throughout existence^8^. From this perspective, tooth loss and maintenance of functional dentition can be understood as markers of life trajectories, reflecting both accumulated inequalities and the ability of public policies to modify these processes over time. Although longitudinal studies are the most appropriate to investigate these trajectories, their performance presents operational and financial limitations^9,10^. Thus, the analysis of series of population-based surveys with repeated cross-sections is an alternative to investigate how different historical contexts experienced throughout life may have had a distinct impact on the maintenance of functional dentition between different age groups, especially considering milestones such as the publication of the National Oral Health Policy (*Política Nacional de Saúde Bucal* – PNSB), in 2004.

In most studies, time trends are described as assuming that the effects over time are homogeneous among age groups. In this study, we assumed that the magnitude of the changes could differ according to the interaction between period and age. Furthermore, we evaluated whether inequalities according to skin color or race remained over the 20 years analyzed.

## METHODS

This is a study of repeated panels that investigated trends and disparities in the presence of functional dentition in the Brazilian population, according to data from SB Brasil. The analysis period covers 20 years, using three sectional studies carried out in the years 2003, 2010, and 2023. Each edition of the survey followed a probabilistic sampling plan stratified by clusters, with representativeness for the capitals and small municipalities in the five geographic regions of the country (North, Northeast, Midwest, Southeast, and South).

The SB Brasil surveys used multistage cluster probabilistic sampling plans to generate estimates for index-age and age groups recommended by the World Health Organization (WHO)^3^. The strategy of geographic stratification varied between cycles. In 2003, stratification was based on the five macroregions and municipality size. Subsequently, in 2010, 32 geographic domains were defined, covering the capitals and small municipalities by region. Finally, in 2023, the plan was redefined to 53 geographic domains, which considered the capitals and states altogether (including capitals and small municipalities).

The eligibility criteria for this analysis were restricted to participants who belonged to the age groups of adults (35–44 years) and older people (65–74 years) present in the three surveys. The sample totaled 54,933 participants, 32,229 adults and 22,704 older adults. The total of participants with complete data included in the final analysis was 54,560 individuals. The percentage of losses due to missing data was 0.68%, which is deemed negligible, and there was no need to apply imputation techniques.

Data collection included household interviews (with structured questionnaire) and oral examinations. The interviews addressed sociodemographic variables such as sex, skin color or race, level of education, and family income. Oral examinations in the three editions of SB Brasil followed a standardized methodology. The teams, composed of dentists and trained notetakers, performed the examinations in the homes using portable equipment. The calibration of the teams was maintained in each cycle through training workshops focusing on consensus among the examiners. An important methodological difference was the technological evolution in 2023 with the incorporation of digital technologies, such as intraoral cameras, aiming for greater diagnostic accuracy^5^.

The outcome variable for this study was the presence of functional dentition. This outcome was developed based on the DMFT index (number of decayed, missing, and filled teeth), using the variable "total number of missing permanent teeth." This variable was recoded, and the number of present teeth was obtained by subtracting the component of missing permanent teeth from 32, based on the DMFT index, which records teeth lost due to dental caries. Functional dentition was defined according to the WHO, considering the presence of at least 20 natural teeth—and which is a widely accepted indicator of maintenance of masticatory function and oral health-related quality of life^3^.

Explanatory variables were considered age (35–39, 40–44, 65–69, and 70–74 years); survey period (2003, 2010, and 2023); and skin color or race, analyzed in the five categories of the Brazilian Institute of Geography and Statistics (IBGE), namely: white, Black, mixed-race, Asian, and Indigenous.

The possibility of estimating cohort effects was initially considered, but the structure of SB Brasil data made their estimation unfeasible (Supplementary Material). The index-age groups of the SB Brasil are discontinuous, and the surveys were carried out at irregular intervals, making it impossible to share years of birth between age and period combinations.

The models of this study included the main effects of age and period as well as the term of interaction between these variables. Age-period interaction was estimated by quasi-Poisson regression, considering the complex sample design and adopting as a reference category the individuals aged 35–39 years in the 2003 survey. Prevalence ratios (PR) higher than 1 indicate that the temporal variation in prevalence in that age group was higher than expected by the main effects of age and period, while values below 1 indicate a variation lower than expected.

Weighted prevalence of functional dentition was estimated by survey and age group, with 95% confidence intervals (95%CI). Two quasi-Poisson regression models with logarithmic binding function were adjusted, providing PR. Model 1 included age, period, and the term of interaction between both. Model 2 added skin color or race, taking the white category as a reference. The significance of the interaction and skin color or race was evaluated by Wald test adjusted for the complex sample design. Variance was estimated by Taylor series linearization with sandwich estimator. Strata with single primary unit were processed by adjustment centered on the sample mean. The analyses were conducted in the R software version 4.3, with the *survey* package, and incorporated the complex sampling design of the three surveys, with selection strata, primary sampling units, and sample weights.

This study is waived from appreciation by the Research Ethics Committee, as secondary, public-domain data are exclusively used. The study was conducted in accordance with the ethical principles of the Declaration of Helsinki and followed the Brazilian ethical standards applicable to secondary data (Resolutions No. 466/2012 and No. 510/2016 of the National Health Council). Raw data are available upon request on the official website of the Brazilian Ministry of Health (https://www.gov.br/saude/pt-br/composicao/saps/brasil-sorridente/sb-brasil/dados).

### Data Availability Statement:

The entire dataset that supports the results of this study is available upon request to the corresponding author. The dataset is not publicly available due to the integration of databases created by the authors.

## RESULTS

The prevalence of functional dentition progressively increased in Brazil between 2003 and 2023, from 52.5% (95%CI 48.8–56.1) in 2003 to 57.2% (95%CI 54.8–59.5) in 2010 and 73.6% (95%CI 71.4–75.8) in 2023 (Table 1). The increase was observed in all age groups. The highest prevalence values were recorded among adults aged 35–39 years in all surveys, while the older adult groups presented the lowest prevalence values, despite the highest relative increases over the period.

**Table 1:** Prevalence of functional dentition according to age group and survey year. Brazil, 2003 to 2023.

Age group (years)	2003	2010	2023
35 to 39	71.5 (68.9–74.2)	86.6 (84.5–88.6)	96.1 (94.4–97.7)
40 to 44	53.7 (48.4–58.9)	72.3 (69.2–75.4)	89.5 (86.3–92.6)
65 to 69	14.9 (11.7–18.1)	13.6 (10.9–16.3)	32.5 (28.5–36.5)
70 to 74	9.3 (5.7–12.9)	13.4 (10.0–16.8)	23.7 (19.4–27.9)
Total	52.5 (48.8–56.1)	57.2 (54.8–59.5)	73.6 (71.4–75.8)

Prevalence values in percentage, with 95% confidence intervals, estimated under the complex sample design.

Source: SB Brasil 2003, 2010, and 2023.

The interaction between age and period was statistically significant (F=12.59; p<0.001), indicating that the temporal evolution of functional dentition occurred differently between age groups (Table 2, Figure 1). Compared to 35–39-year-old individuals in 2003, the prevalence was progressively lower among the older age groups, reflecting the strong association between aging and lower presence of functional dentition. However, the interaction terms regarding 2023 were positive and increased according to age, with greater magnitude in the older adult groups.

**Table 2:** Prevalence ratios of functional dentition according to age, period, and skin color or race. Brazil, 2003 to 2023.

			Model 1	Model 2
Term				
	Age (reference: 35 to 39 years)			
		40 to 44	0.75 (0.68–0.83)	0.75 (0.68–0.82)
		65 to 69	0.21 (0.17–0.26)	0.21 (0.17–0.26)
		70 to 74	0.13 (0.09–0.19)	0.13 (0.09–0.19)
	Period (reference: 2003)			
		2010	1.21 (1.16–1.26)	1.20 (1.15–1.26)
		2023	1.34 (1.29–1.40)	1.35 (1.29–1.41)
	Age-period interaction			
		40–44×2010	1.11 (1.00–1.24)	1.12 (1.01–1.23)
		65–69×2010	0.75 (0.57–1.01)	0.76 (0.57–1.01)
		70–74×2010	1.19 (0.75–1.90)	1.20 (0.75–1.91)
		40–44×2023	1.24 (1.12–1.38)	1.24 (1.12–1.36)
		65–69×2023	1.62 (1.27–2.08)	1.63 (1.27–2.08)
		70–74×2023	1.90 (1.23–2.92)	1.90 (1.23–2.93)
	Skin color or race (reference: white)			
		Black	—	0.95 (0.91–0.99)
		Mixed-race	—	0.91 (0.88–0.94)
		Asian	—	0.93 (0.85–1.02)
		Indigenous	—	0.98 (0.81–1.20)

Prevalence ratios and 95% confidence intervals, estimated by quasi-Poisson regression under the complex sample design.

Model 1: n=54,557; scattering parameter=0.388. Model 2: n=54,287, considering that 270 records had no information on skin color or race; scattering parameter=0.387.

Source: SB Brasil 2003, 2010, and 2023.

**Figure 1:**
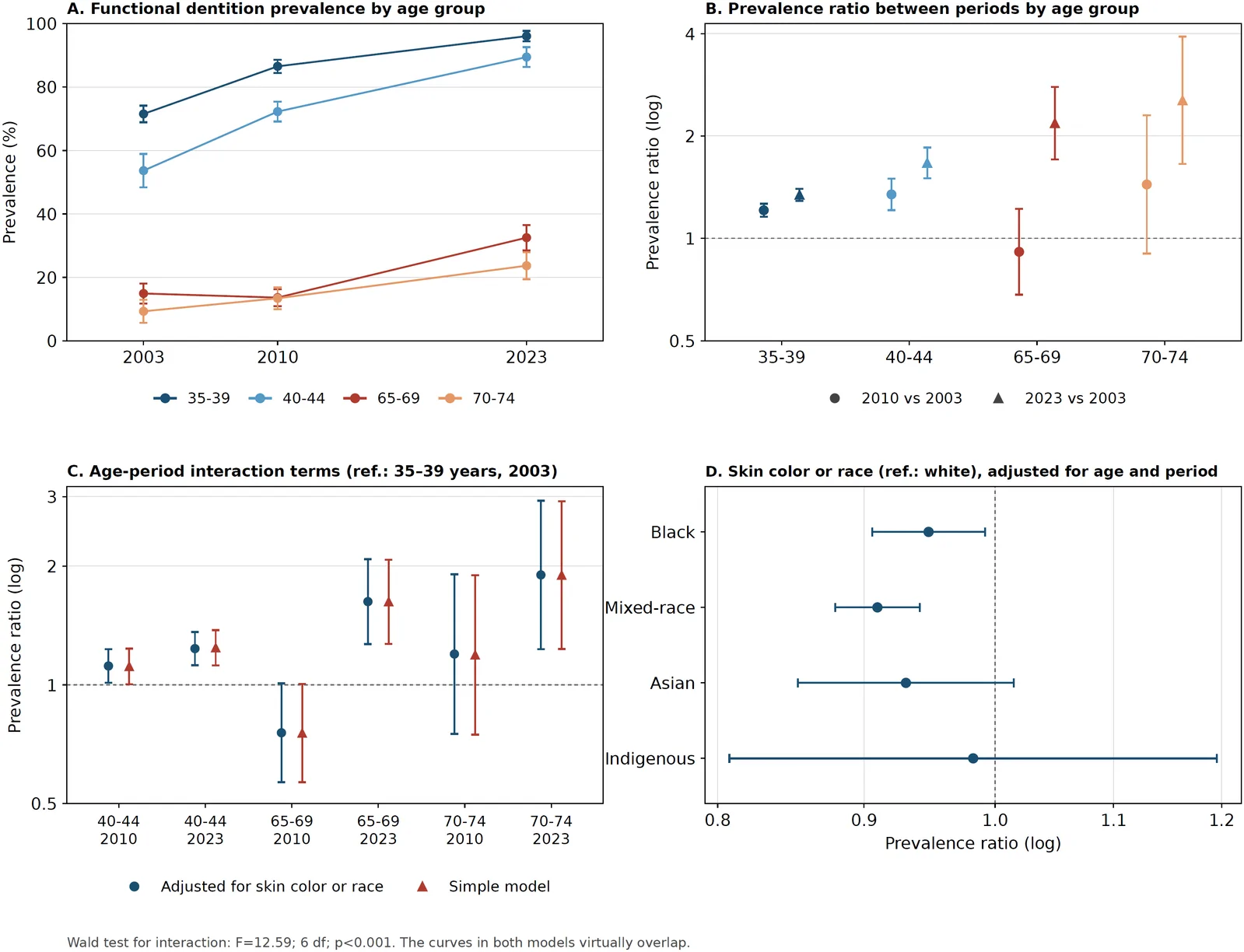
Prevalence and prevalence ratios of functional dentition between periods by age group. Terms of interaction between age and period. Model for skin color or race adjusted for age and period, Brazil, 2003–2023.

A) Dentition prevalence by age group; B) Prevalence ratio between periods by age group; C) Age-period interaction terms, considering 35–39 years and 2003 as references. The red lines represent the simple model, and the blue lines indicate the model adjusted for skin color or race; D) Prevalence ratio by skin color or race adjusted for age x period, considering the white skin color or race as reference.

Source: SB Brasil 2003, 2010, and 2023.

Differences according to skin color or race remained after adjustment for age, period, and age-period interaction (Table 2). We observed lower prevalence of functional dentition among mixed-race (PR 0.91; 95%CI 0.88–0.94) and Black people (PR 0.95; 95%CI 0.91–0.99) compared to white people. We verified no statistically significant differences between Asian (PR 0.93; 95%CI 0.85–1.02) and Indigenous people (PR 0.98; 95%CI 0.81–1.20). The study, however, is limited by the low representativeness of both groups in the sample.

The inclusion of skin color or race in the multivariate model did not substantially modify the estimates of age, period, or age-period interaction effects. The prevalence ratios slightly differed between the models, and the significance of the interaction remained unchanged (F=12.59), indicating that the inclusion of race or skin color did not change the difference in temporal trajectories observed between age groups. Nevertheless, significant differences in prevalence remained, with persistent effect over the years.

## DISCUSSION

Unlike most national investigations, which describe temporal trends through comparisons between age-stratified surveys or analyses, in this study, we directly investigated whether the magnitude of changes occurring over time differed between age groups. The main contribution of this study is not only to demonstrate improvements in functional dentition, but to show that the effects associated with the historical period depended on the age of the individuals, indicating that population changes occurred between 2003 and 2023 produced different responses according to the analyzed generation and evidencing a pattern that would hardly be captured by analyses based only on the isolated effects of these dimensions. The results suggest that improvements over the last two decades have not benefited all generations alike.

The increase in the prevalence of functional dentition observed during the analyzed period coincides with important changes in the oral health scenario and social conditions in Brazil. Among these changes, we highlight the expansion of dental care in the Brazilian Unified Health System (*Sistema Único de Saúde –* SUS) after the implementation of PNSB^11^; the increase in level of education^12^; the expansion of social protection programs, such as *Bolsa Família* (Brazilian cash transfer program)^13^; the strengthening of health promotion initiatives^14^; and the economic transformations observed during the 2000s, followed by periods of deceleration^15^. Although the design of this study does not allow the observed gains to be attributed to any of these changes alone, the findings are compatible with the hypothesis that the evolution of functional dentition occurred in a context of social and institutional transformations that, together, may have favored better oral health conditions for the Brazilian population.

The greater preservation of dentition observed in this study follows a trend described globally. Authors of studies conducted in England^16^, Japan^17^, the United States^18^, and China^19^ documented progressive improvement in dental maintenance among generations, with different magnitudes among cohorts, widely attributed to changes in living conditions, increased access to dental services, and reduced edentulism. Part of this literature focused on the description of the effects of age, period, or cohort (APC) for investigating the scenario; moreover, the results of the present study dialogue with the aforementioned studies, considering that the methodology of interaction between age and period allowed us to identify heterogeneity of period effects.

Isolated effects of age confirm the reduction of the maintenance of functional dentition with the advance of aging, reflecting both the biological process and the accumulation of social disadvantages throughout life^20^. In addition, the age and period interaction evidences the lower prevalence of functional dentition and higher relative gains regarding the prevalence of the outcome in older adults. This pattern suggests that improvements in the social context and oral health care may have later repercussions in older adults, considering that this population is greatly affected by the social gradient, translated into complex barriers to be faced. From the perspective of healthy aging^21^, these findings reinforce that tooth loss should be understood not only as a clinical outcome, but as a factor of impairment of oral function capacity, with potential limitation of chewing, communication, feeding, and social participation^22^. These results point to the need for care strategies that consider the different needs of the stages of life, with special attention to the preservation of oral functionality of older adults.

According to the findings, policies implemented at a given historical moment do not restart oral health trajectories; they act on individuals who accumulate very distinct exposures throughout their lives. Therefore, people evaluated in the same year respond differently to the same social changes. The observed pattern is compatible with the perspective of the course of life, according to which oral health results from the accumulation of biological, social, and care exposures over time^8^. Older adults, in addition to accumulating longer exposure to social determinants of health, grew up in a period marked by lower access to dental care, absence of preventive policies, and persistent social inequalities, while younger adults were benefited by the expansion of fluoridation, oral health care, and preventive measures, even concerning the consumption of harmful substances such as tobacco. Conversely, younger generations have come to live more intensely with structured systems, which coexist with new risk factors, such as increased consumption of ultra-processed foods and sugary drinks, associated with obesity, metabolic diseases, and worse outcomes in oral health^23-30^.

Thus, individuals evaluated in the same historical period do not necessarily share the same opportunities for dental preservation, since they belong to generations who have experienced very distinct contexts of prevention, treatment, and organization of oral health services^31-36^. The greatest relative gains observed among older adults suggest that recent transformations have changed historically unfavorable trajectories, although they have not eliminated the accumulated disadvantages, resulting in the beginning of a transition of the epidemiological profile of oral health of Brazilians, in which aging progressively ceases to be inevitably associated with edentulism.

The adjustment for skin color or race produced only minor changes in the effects of age and period. In addition, we perceived a consistent difference in the prevalence of functional dentition among racial groups throughout the analyzed period. Considering this finding, racial inequalities do not result from changes in the age composition of the population or from temporal transformations perceived by the period effect, but represent a persistent and lasting effect on oral health. In other words, the advances observed in the last two decades occurred in all groups, but were not enough to substantially reduce the difference in the prevalence of functional dentition among white, Black, and mixed-race people, reinforcing the permanence of the structure of iniquity regarding the outcome. As for the evaluation of the PNSB and the policies for mitigating iniquities, we verified an important aspect in its actions and services: they may be centered on the reduction of the problems themselves, leaving the reduction of iniquities in the background.

This result is consistent with previous research according to which there are worse oral health indicators among Black people, including higher prevalence of edentulism, higher occurrence of untreated caries, and lower access to restorative procedures, even after considering socioeconomic differences^37,38^. The persistence of these inequalities suggests that the observed differences cannot be understood only by individual characteristics, but are related to historical and structural processes.

Data from the 2019 National Survey of Health (*Pesquisa Nacional de Saúde* – PNS) reinforce this interpretation by demonstrating that Black and mixed-race adults are more likely to not have had a dental consultation in the last year, even after adjustments for region and income^23^. According to these findings, skin color or race remains associated with inequalities in access to and use of dental services, reflecting mechanisms related to institutional discrimination and unequal distribution of resources and opportunities for care. A similar pattern has been described in different international contexts, including the United States^39^, the United Kingdom^40^, and Canada^41^, indicating that racial inequalities in health may persist even in structured health systems.

Among the strengths of this study are the use of national oral health surveys carried out in different historical periods and the application of an analytical approach capable of investigating how age and period relate to the evolution of functional dentition. The analysis of repeated cross-section cohorts allowed us to evaluate population changes over two decades and to identify that the observed gains did not occur uniformly among age groups. Furthermore, the incorporation of skin color or race made it possible to analyze the persistence of social inequalities in an outcome relevant to oral health and healthy aging.

Some limitations should be considered, as these are data from repeated cross-sectional surveys. It was not possible to follow up individuals over time or to completely distinguish the effects related to the cohort, only age and period. Even if the interaction between age and period allows for the investigation of differences between age groups at different historical moments, their results should be interpreted considering the limitations inherent in observational study designs. In addition, the measurement of functional dentition based on the presence of 20 or more remaining teeth^3^, widely used in epidemiological studies, does not incorporate specific clinical and functional aspects such as the presence of occlusal pairs, distribution of remaining teeth, need for treatment, or functional impact of dental condition.

The findings suggest that Brazilian oral health is undergoing a paradigm shift. New generations tend to age maintaining an increasing number of natural teeth, imposing new challenges to dental services, which begin to serve older adults with different needs from those observed in previous generations. This transition, however, occurs unevenly; while gains between generations can evidence the impact of social and welfare improvements accumulated in recent decades, the persistence of racial differences shows that population progress alone is not enough to reduce structural inequalities. This suggests that policies oriented predominantly toward the improvement of the average health indicators can produce important advances, without focusing on the reduction of structural inequalities. The challenge is not only to increase the dental longevity of the population, but to ensure that the policy action is a proxy of the mitigation of inequalities.
