# Supplementary material for: Effects of age and period on functional dentition: an analysis of skin color/race inequalities in the 2003–2023 National Oral Health Surveys (SB Brasil)
Source: Rev Bras Epidemiol. 2026 Oct 19;29(Suppl 1):e260033supl1. doi: 10.1590/1980-549720260033.supl.1 (PMC13679413; doi:10.1590/1980-549720260033.supl.1)
Supplement: Supplementary file 1 [file 1980-5497-rbepid-29-suppl1-e260033supl1-Suppl01.pdf]

**Material Suplementar**

Coortes de nascimento nas combinações de idade e período dos Inquéritos Nacionais de Saúde Bucal (SBBrasil) e razão pela qual os termos de interação não podem ser lidos como efeitos de coorte.

**Parte A.** Coortes de nascimento observadas em cada combinação de idade e períodos. Brasil 2003 a 2023.

| Grupo etário | 2003 | 2010 | 2023 |
|---|---|---|---|
| 35 a 39 anos | 1964 a 1968 | 1971 a 1975 | 1984 a 1988 |
| 40 a 44 anos | 1959 a 1963 | 1966 a 1970 | 1979 a 1983 |
| 65 a 69 anos | 1934 a 1938 | 1941 a 1945 | 1954 a 1958 |
| 70 a 74 anos | 1929 a 1933 | 1936 a 1940 | 1949 a 1953 |

Coorte de nascimento igual ao ano do inquérito menos a idade. Apenas duas coortes aparecem em mais de uma combinação: os nascidos entre 1934 e 1940, observados aos 65 a 69 anos em 2003 e aos 70 a 74 anos em 2010; e os nascidos entre 1964 e 1970, observados aos 35 a 39 anos em 2003 e aos 40 a 44 anos em 2010. Nenhuma combinação do inquérito de 2023 compartilha ano de nascimento com os inquéritos anteriores.

**Parte B.** Coortes atribuídas pelo procedimento de diagonais dos modelos idade-período-coorte. Brasil 2003 a 2023.

| Coorte atribuída | Combinações reunidas | Anos de nascimento efetivos |
|---|---|---|
| 1 | 70-74 anos em 2003 | 1929 a 1933 |
| 2 | 65-69 em 2003 e 70-74 em 2010 | 1934 a 1940 |
| 3 | 40-44 em 2003, 65-69 em 2010 e 70-74 em 2023 | 1959-1963; 1941-1945; 1949-1953 |
| 4 | 35-39 em 2003, 40-44 em 2010 e 65-69 em 2023 | 1964-1968; 1966-1970; 1954-1958 |
| 5 | 35-39 em 2010 e 40-44 em 2023 | 1971-1975; 1979-1983 |
| 6 | 35-39 anos em 2023 | 1984 a 1988 |

As coortes 3 e 4 reúnem combinações de adultos e de idosos que não compartilham qualquer ano de nascimento. Nessas duas coortes, o desvio estimado corresponde à diferença de prevalência entre adultos e idosos, isto é, a um efeito de idade, e não a uma experiência geracional compartilhada.
