# Supplementary material for: Effects of age and period on functional dentition: an analysis of skin color/race inequalities in the 2003–2023 National Oral Health Surveys (SB Brasil)
Source: Rev Bras Epidemiol. 2026 Oct 19;29(Suppl 1):e260033supl1. doi: 10.1590/1980-549720260033.supl.1 (PMC13679413; doi:10.1590/1980-549720260033.supl.1)
Supplement: Translation

# Translation: Efeitos de idade e período na dentição funcional: análise das desigualdades por cor da pele ou raça nos Inquéritos Nacionais de Saúde Bucal 2003-2023

**DOI:** 10.1590/1980-549720260033.supl.1.2

**Authors:** Maria Helena Rodrigues Galvão, Sophia Queiroz Marques dos Santos, Niely Enetice de Sousa Catão, Ane Polline Lacerda Protasio

**Affiliations:** I Universidade Federal de Pernambuco – Vitória de Santo Antão (PE), Brasil.; II Universidade Federal do Rio Grande do Norte – Natal (RN), Brasil.; III Universidade Federal da Paraíba – João Pessoa (PB), Brasil.

**Keywords:** Inquéritos de saúde, Saúde bucal, Perda dentária, Análise de dados secundários, Ciclo de vida

## Abstract

**Objetivo:**

Estimar interação entre idade e período na manutenção da dentição funcional entre adultos e idosos brasileiros em 2003, 2010 e 2023 e investigar desigualdades segundo cor da pele ou raça.

**Métodos:**

Foram analisados dados dos Inquéritos Nacionais de Saúde Bucal de 2003, 2010 e 2023, compondo uma série temporal de cortes transversais repetidos com 54.557 participantes de 35–44 e 65–74 anos. Foram estimadas Razões de Prevalência (RP) por regressão quasi-Poisson, contendo termos para idade, período e interação idade e período, com e sem ajuste para cor da pele ou raça, considerando o desenho amostral complexo.

**Resultados:**

Interação idade e período foi significativa (p<0,001), indicando que mudanças temporais diferiram entre grupos etários. A prevalência de dentição funcional aumentou de 52,5% (IC95% 48,8–56,1) em 2003 para 73,6% (IC95% 71,4–75,8) em 2023. Entre adultos, aumentou de 71,5 para 96,1%, enquanto entre idosos houve redução entre 2003 e 2010, embora em 2023 tenha sido mais que o dobro da observada em 2003. Termos de interação referentes a 2023 foram positivos e aumentaram conforme a idade. Após ajuste por cor da pele ou raça, indivíduos pardos (RP 0,91; IC95% 0,88–0,94) e pretos (RP 0,95; IC95% 0,91–0,99) apresentaram menor prevalência de dentição funcional em comparação aos brancos, sem diferenças significativas para os demais.

**Conclusão:**

A manutenção da dentição funcional apresentou aumento expressivo entre 2003 e 2023, porém com trajetórias distintas entre adultos e idosos, evidenciando avanços tardios na população idosa. Desigualdades segundo cor da pele ou raça persistiram, afetando principalmente pessoas pretas e pardas.

## INTRODUÇÃO

A perda dentária constitui um dos desfechos mais graves em saúde bucal e reflete a exposição acumulada a condições sociais, econômicas, comportamentais e ao acesso aos serviços de saúde ao longo da vida^1,2^. Entre seus indicadores, a dentição funcional, que pode ser definida pela presença de pelo menos 20 dentes naturais^3^, representa um desfecho de maior relevância para a saúde pública por preservar funções mastigatórias, fonéticas, estéticas e sociais, estando associada à qualidade de vida e ao envelhecimento saudável^1,2^.

Os Inquéritos Nacionais de Saúde Bucal (SBBrasil) mostram melhora progressiva das condições de saúde bucal da população brasileira nas últimas décadas, evidenciada pela redução do edentulismo e pelo aumento da preservação dentária. Essas mudanças ocorreram em um contexto de transformações sociais e de ampliação das políticas públicas de saúde bucal, entretanto, seus benefícios podem não ter sido distribuídos de forma homogênea entre diferentes segmentos populacionais^4-6^.

No Brasil, os estudos sobre perda dentária e dentição funcional têm se baseado predominantemente em inquéritos transversais de base populacional. As evidências mostram persistência de desigualdades segundo cor da pele ou raça, renda e escolaridade, com menor prevalência de dentição funcional entre pessoas negras e grupos socialmente desfavorecidos, apesar da melhoria observada nos indicadores populacionais^4,5,7^. Permanece, todavia, pouco explorado se essa evolução ocorreu de maneira semelhante entre adultos e idosos, e se os efeitos das mudanças observadas ao longo do tempo sobre a dentição funcional diferiram entre os grupos etários.

Essa questão é particularmente relevante à luz da epidemiologia do curso da vida, segundo a qual a saúde na idade adulta e na velhice resulta do acúmulo de exposições biológicas, sociais e materiais ao longo da existência^8^. Nessa perspectiva, a perda dentária e a manutenção da dentição funcional podem ser compreendidas como marcadores de trajetórias de vida, refletindo tanto desigualdades acumuladas quanto a capacidade das políticas públicas de modificar esses processos ao longo do tempo. Embora estudos longitudinais sejam os mais adequados para investigar essas trajetórias, sua realização apresenta limitações operacionais e financeiras^9,10^. Assim, a análise de séries de inquéritos populacionais com cortes transversais repetidos constitui uma alternativa para investigar como diferentes contextos históricos vivenciados ao longo da vida podem ter repercutido de forma distinta na manutenção da dentição funcional entre diferentes grupos etários, sobretudo considerando marcos como a publicação da Política Nacional de Saúde Bucal (PNSB), em 2004.

A maior parte dos estudos descreve tendências temporais assumindo que os efeitos ao longo do tempo são homogêneos entre grupos etários. Neste estudo, partiu-se da hipótese de que a magnitude das mudanças poderia diferir conforme a interação entre período e idade. Adicionalmente, avaliou-se se as desigualdades segundo cor da pele ou raça permaneceram ao longo dos 20 anos analisados.

## MÉTODOS

Trata-se de um estudo de painéis repetidos que investigou as tendências e disparidades na presença de dentição funcional na população brasileira, de acordo com dados do SBBrasil. O período de análise abrange 20 anos, utilizando 3 estudos seccionais realizados nos anos de 2003, 2010 e 2023. Cada edição da pesquisa seguiu um plano amostral probabilístico estratificado e por conglomerados, com representatividade para as capitais e municípios do interior nas cinco regiões geográficas do país (Norte, Nordeste, Centro-Oeste, Sudeste e Sul).

Os inquéritos SBBrasil utilizaram planos amostrais probabilísticos por conglomerados em múltiplos estágios com o objetivo de gerar estimativas para idades-índice e grupos etários preconizados pela Organização Mundial de Saúde (OMS)^3^. A estratégia de estratificação geográfica variou entre os ciclos. Em 2003, a estratificação se baseou nas cinco macrorregiões e no porte do município. Posteriormente, em 2010, foram definidos 32 domínios geográficos, abrangendo as capitais e os municípios do interior por região. Por fim, em 2023, o plano foi redefinido para 53 domínios geográficos, que consideraram as capitais e os estados como um todo (incluindo capitais e municípios do interior).

Os critérios de elegibilidade para esta análise foram restritos aos participantes que pertenciam aos grupos etários de adultos (35 a 44 anos) e pessoas idosas (65 a 74 anos) presentes nos 3 inquéritos. A amostra totalizou 54.933 participantes, sendo 32.229 adultos e 22.704 pessoas idosas. O total de participantes com dados completos incluídos na análise final foi de 54.560 pessoas. O percentual de perdas por dados omissos foi de 0,68%, que é considerado desprezível, e não houve necessidade de aplicação de técnicas de imputação.

A coleta de dados incluiu entrevistas domiciliares (com questionário estruturado) e exames odontológicos. As entrevistas abordaram variáveis sociodemográficas como sexo, cor da pele ou raça, escolaridade e renda familiar. Os exames bucais nas três edições do SBBrasil seguiram uma metodologia padronizada. As equipes, compostas por cirurgiões-dentistas e anotadores treinados, realizaram os exames nos domicílios utilizando equipamentos portáteis. A calibração das equipes foi mantida em cada ciclo por meio de oficinas de treinamento com foco no consenso entre os examinadores. Uma diferença metodológica importante foi a evolução tecnológica em 2023 com a incorporação de tecnologias digitais, como câmeras intraorais, visando maior precisão diagnóstica^5^.

A variável desfecho para este estudo foi a presença de dentição funcional. Este desfecho foi construído a partir do Índice CPO-D (número de dentes cariados, perdidos e obturados), utilizando a variável "número total de dentes permanentes perdidos". Esta variável foi recodificada, e o número de dentes presentes foi obtido subtraindo-se de 32 o componente de dentes permanentes perdidos do índice CPO-D, que registra as perdas por cárie. Foi adotada a definição de dentição funcional pela Organização Mundial de Saúde (OMS), que exige a presença de pelo menos 20 dentes naturais — e que é um indicador amplamente aceito de manutenção da função mastigatória e qualidade de vida oral^3^.

Como variáveis explicativas, foram consideradas a idade (35–39, 40–44, 65–69 e 70–74 anos); o período do inquérito (2003, 2010 e 2023); e a cor da pele ou raça, analisada nas cinco categorias do Instituto Brasileiro de Geografia e Estatística (IBGE): branca, preta, parda, amarela e indígena.

A possibilidade de estimar efeitos de coorte foi inicialmente considerada, mas a estrutura dos dados do SBBrasil impossibilitou sua estimação (Material Suplementar). As faixas etárias-índice do SBBrasil são descontínuas, e os inquéritos foram realizados em intervalos irregulares, impossibilitando o compartilhamento de anos de nascimento entre as combinações de idade e período.

Os modelos deste estudo incluíram os efeitos principais de idade e período, bem como o termo de interação entre essas variáveis. A interação idade-período foi estimada por regressão quasi-Poisson, considerando o desenho amostral complexo e adotando como categoria de referência os indivíduos de 35–39 anos no inquérito de 2003. Razões de Prevalência (RP) superiores a 1 indicam que a variação temporal da prevalência naquele grupo etário foi maior do que a esperada pelos efeitos principais de idade e período, enquanto valores inferiores a 1 indicam variação menor que a esperada.

Foram estimadas as prevalências ponderadas de dentição funcional por inquérito e por grupo etário, com Intervalos de Confiança de 95% (IC95%). Dois modelos de regressão quasi-Poisson com função de ligação logarítmica foram ajustados, fornecendo RP. O modelo 1 incluiu idade, período e o termo de interação entre ambos. O modelo 2 acrescentou a cor da pele ou raça, tomando a categoria branca como referência. A significância da interação e da cor da pele ou raça foi avaliada por teste de Wald ajustado ao desenho amostral complexo. A variância foi estimada por linearização de Taylor com estimador sanduíche. Estratos com unidade primária única foram tratados por ajuste centrado na média da amostra. As análises foram conduzidas no R versão 4.3, com o pacote *survey*, e incorporaram o desenho amostral complexo dos três inquéritos, com estratos de seleção, unidades primárias de amostragem e pesos amostrais.

Esta pesquisa dispensou apreciação pelo Comitê de Ética em Pesquisa, por utilizar exclusivamente dados secundários de domínio público. O estudo foi conduzido em conformidade com os princípios éticos da Declaração de Helsinque e seguiu as normas éticas brasileiras aplicáveis a dados secundários (Resoluções n° 466/2012 e n° 510/2016 do Conselho Nacional de Saúde). Os dados brutos estão disponíveis mediante solicitação no portal oficial do Ministério da Saúde (https://www.gov.br/saude/pt-br/composicao/saps/brasil-sorridente/sb-brasil/dados).

### Declaração de disponibilidade de dados:

Todo o conjunto de dados que dá suporte aos resultados deste estudo está disponível mediante solicitação ao autor correspondente. O conjunto de dados não está publicamente disponível devido à integração de bancos de dados realizados pelos autores.

## RESULTADOS

A prevalência de dentição funcional aumentou progressivamente no Brasil entre 2003 e 2023, passando de 52,5% (IC95% 48,8–56,1) em 2003 para 57,2% (IC95% 54,8–59,5) em 2010 e 73,6% (IC95% 71,4–75,8) em 2023 (Tabela 1). O aumento foi observado em todas as faixas etárias. As maiores prevalências foram registradas entre os adultos de 35–39 anos em todos os inquéritos, enquanto os grupos idosos apresentaram as menores prevalências, apesar dos maiores incrementos relativos ao longo do período.

**Tabela 1:** Prevalência de dentição funcional segundo grupo etário e ano do inquérito. Brasil, 2003 a 2023.

Grupo etário (anos)	2003	2010	2023
35 a 39	71,5 (68,9–74,2)	86,6 (84,5–88,6)	96,1 (94,4–97,7)
40 a 44	53,7 (48,4–58,9)	72,3 (69,2–75,4)	89,5 (86,3–92,6)
65 a 69	14,9 (11,7–18,1)	13,6 (10,9–16,3)	32,5 (28,5–36,5)
70 a 74	9,3 (5,7–12,9)	13,4 (10,0–16,8)	23,7 (19,4–27,9)
Total	52,5 (48,8–56,1)	57,2 (54,8–59,5)	73,6 (71,4–75,8)

Prevalências em porcentagem, com intervalos de confiança de 95%, estimadas sob o desenho amostral complexo.

Fonte: SB Brasil 2003, 2010 e 2023.

A interação entre idade e período foi estatisticamente significativa (F=12,59; p<0,001), indicando que a evolução temporal da dentição funcional ocorreu de maneira diferente entre os grupos etários (Tabela 2, Figura 1). Em comparação aos indivíduos de 35-39 anos em 2003, a prevalência foi progressivamente menor entre as faixas etárias mais elevadas, refletindo a forte associação entre envelhecimento e menor presença de dentição funcional. Entretanto, os termos de interação referentes a 2023 foram positivos e aumentaram conforme a idade, com maior magnitude nos grupos idosos.

**Tabela 2:** Razões de prevalência de dentição funcional segundo idade, período e cor da pele ou raça. Brasil, 2003 a 2023.

			Modelo 1	Modelo 2
Termo				
	Idade (referência: 35 a 39 anos)			
		40 a 44	0,75 (0,68–0,83)	0,75 (0,68–0,82)
		65 a 69	0,21 (0,17–0,26)	0,21 (0,17–0,26)
		70 a 74	0,13 (0,09–0,19)	0,13 (0,09–0,19)
	Período (referência: 2003)			
		2010	1,21 (1,16–1,26)	1,20 (1,15–1,26)
		2023	1,34 (1,29–1,40)	1,35 (1,29–1,41)
	Interação idade-período			
		40-44×2010	1,11 (1,00–1,24)	1,12 (1,01–1,23)
		65-69×2010	0,75 (0,57–1,01)	0,76 (0,57–1,01)
		70-74×2010	1,19 (0,75–1,90)	1,20 (0,75–1,91)
		40-44×2023	1,24 (1,12–1,38)	1,24 (1,12–1,36)
		65-69×2023	1,62 (1,27–2,08)	1,63 (1,27–2,08)
		70-74×2023	1,90 (1,23–2,92)	1,90 (1,23–2,93)
	Cor da pele ou raça (referência: branca)			
		Preta	—	0,95 (0,91–0,99)
		Parda	—	0,91 (0,88–0,94)
		Amarela	—	0,93 (0,85–1,02)
		Indígena	—	0,98 (0,81–1,20)

Razões de prevalência e intervalos de confiança de 95%, estimados por regressão quasi-Poisson sob o desenho amostral complexo.

Modelo 1: n=54.557; parâmetro de dispersão=0,388. Modelo 2: n=54.287, em razão de 270 registros sem informação de cor da pele ou raça; parâmetro de dispersão=0,387.

Fonte: SB Brasil 2003, 2010 e 2023.

**Figura 1:** Prevalência e razões de prevalência da dentição funcional entre períodos por grupo etário. Termos de interação entre idade e período. Modelo para cor da pele ou raça ajustado por idade e período, Brasil, 2003–2023.

A) Prevalência da dentição por grupo etário; B) Razão de prevalência entre períodos por grupo etário; C) Os termos de interação idade-período, sendo 35–39 anos e 2003 as referências. As linhas vermelhas representam o modelo simples, e as linhas azuis indicam o modelo ajustado por cor da pele ou raça; D) Razão de prevalência por cor da pele ou raça ajustada por idade x período, sendo a cor da pele ou raça branca a referência.

Fonte: SB Brasil 2003, 2010 e 2023.

As diferenças segundo cor da pele ou raça permaneceram após o ajuste por idade, período e interação idade-período (Tabela 2). Observou-se menor prevalência de dentição funcional entre pessoas pardas (RP 0,91; IC95% 0,88–0,94) e pretas (RP 0,95; IC95% 0,91–0,99) em comparação às pessoas brancas. Não foram identificadas diferenças estatisticamente significativas entre pessoas amarelas (RP 0,93; IC95% 0,85–1,02) e indígenas (RP 0,98; IC95% 0,81–1,20). O estudo, entretanto, limita-se pela baixa representatividade de ambos os grupos na amostra.

A inclusão da cor da pele ou raça no modelo multivariado não modificou substancialmente as estimativas dos efeitos de idade, período ou da interação idade-período. As razões de prevalência diferiram minimamente entre os modelos, e a significância da interação permaneceu inalterada (F=12,59), indicando que a inclusão da raça ou cor da pele não modificou a diferença das trajetórias temporais observadas entre os grupos etários. Apesar disso, manteve diferenças significativas na prevalência, com efeito persistente ao longo dos anos.

## DISCUSSÃO

Diferentemente da maior parte das investigações nacionais, que descrevem tendências temporais por meio de comparações entre inquéritos ou análises estratificadas por idade, este estudo investigou diretamente se a magnitude das mudanças ocorridas ao longo do tempo diferiu entre os grupos etários. A principal contribuição deste estudo não reside apenas em demonstrar melhora da dentição funcional, mas em evidenciar que os efeitos associados ao período histórico dependeram da idade dos indivíduos, indicando que mudanças populacionais ocorridas entre 2003 e 2023 produziram respostas distintas conforme a geração analisada e revelando um padrão que dificilmente seria captado por análises baseadas apenas nos efeitos isolados dessas dimensões. Os resultados sugerem que as melhorias ocorridas nas últimas duas décadas não beneficiaram igualmente todas as gerações.

O aumento da prevalência de dentição funcional observado ao longo do período analisado coincide com importantes transformações no cenário da saúde bucal e das condições sociais no Brasil. Entre essas mudanças, destacam-se a expansão da atenção odontológica no Sistema Único de Saúde (SUS) após a implementação da PNSB^11^; o aumento da escolarização^12^; a ampliação de programas de proteção social, como o Bolsa Família^13^; o fortalecimento de ações de promoção da saúde^14^; e as transformações econômicas observadas ao longo dos anos 2000, seguidas por períodos de desaceleração^15^. Embora o delineamento deste estudo não permita atribuir os ganhos observados a qualquer uma dessas mudanças de forma isolada, os achados são compatíveis com a hipótese de que a evolução da dentição funcional ocorreu em um contexto de transformações sociais e institucionais que, em conjunto, podem ter favorecido melhores condições de saúde bucal na população brasileira.

A maior preservação da dentição observada neste estudo acompanha uma tendência descrita globalmente. Estudos conduzidos na Inglaterra^16^, Japão^17^, Estados Unidos^18^ e China^19^ documentaram melhora progressiva na manutenção dentária entre gerações, com diferentes magnitudes entre coortes, amplamente atribuída às mudanças nas condições de vida, à ampliação do acesso aos serviços odontológicos e à redução do edentulismo. Parte dessa literatura se concentrou na descrição dos efeitos de idade, período ou coorte (APC) para investigação do cenário; ademais, os resultados do presente estudo dialogam com o exposto, tendo em vista que a metodologia da interação entre idade e período permitiu identificar heterogeneidade dos efeitos de período.

Efeitos isolados da idade confirmam a redução da manutenção da dentição funcional com o avanço do envelhecimento, refletindo tanto o processo biológico quanto a acumulação de desvantagens sociais ao longo da vida^20^. Ademais, a interação idade e período revela as menores prevalências de dentição funcional e maiores ganhos relativos quanto à prevalência do desfecho nos idosos. Esse padrão sugere que melhorias no contexto social e na atenção à saúde bucal podem ter repercutido mais tardiamente nos idosos, tendo em vista que essa parcela da população tem grande potencialização do gradiente social, traduzido em barreiras complexas a serem combatidas. Sob a perspectiva do envelhecimento saudável^21^, esses achados reforçam que a perda dentária deve ser compreendida não apenas como um desfecho clínico, mas como um fator de comprometimento da capacidade da função oral, com potencial limitação da mastigação, comunicação, alimentação e participação social^22^. Esses resultados alertam para a necessidade de estratégias de cuidado que considerem as diferentes necessidades das fases da vida, com atenção especial à preservação da funcionalidade oral da pessoa idosa.

Os achados evidenciam que políticas implementadas em determinado momento histórico não reiniciam as trajetórias de saúde bucal, elas atuam sobre indivíduos que acumulam exposições muito distintas ao longo da vida. Por isso, pessoas avaliadas no mesmo ano respondem de maneira diferente às mesmas mudanças sociais. O padrão observado é compatível com a perspectiva do curso da vida, segundo a qual a saúde bucal resulta da acumulação de exposições biológicas, sociais e assistenciais ao longo do tempo^8^. Os idosos, além de acumularem maior tempo de exposição aos determinantes sociais da saúde, cresceram em um período marcado por menor acesso à atenção odontológica, ausência de políticas preventivas e desigualdades sociais persistentes, enquanto adultos mais jovens foram beneficiados pela expansão da fluoretação, da atenção em saúde bucal e de medidas preventivas, até mesmo quanto ao consumo de substâncias prejudiciais como o tabaco. Em contrapartida, gerações mais recentes passaram a conviver mais intensamente com sistemas estruturados, que coexistem com novos fatores de risco, como o aumento do consumo de alimentos ultraprocessados e bebidas açucaradas, associados à obesidade, doenças metabólicas e piores desfechos em saúde bucal^23-30^.

Assim, indivíduos avaliados em um mesmo período histórico não compartilham necessariamente as mesmas oportunidades de preservação dentária, pois pertencem a gerações que experimentaram contextos muito distintos de prevenção, tratamento e organização dos serviços odontológicos^31-36^. Os maiores ganhos relativos observados entre idosos sugerem que transformações recentes passaram a modificar trajetórias historicamente desfavoráveis, ainda que não tenham eliminado as desvantagens acumuladas, traduzindo-se no início de uma transição do perfil epidemiológico de saúde bucal dos brasileiros, na qual o envelhecimento deixa progressivamente de estar associado de forma inevitável ao edentulismo.

O ajuste por cor da pele ou raça produziu apenas pequenas alterações nos efeitos de idade e período, ademais, uma diferença consistente na prevalência de dentição funcional entre os grupos raciais é percebida ao longo de todo o período analisado. Esse achado indica que as desigualdades raciais não decorrem de mudanças na composição etária da população nem das transformações temporais capturadas pelo efeito de período, mas representam um efeito persistente e duradouro sobre a saúde bucal. Em outras palavras, os avanços observados nas últimas duas décadas ocorreram em todos os grupos, porém não foram suficientes para reduzir substancialmente a diferença na prevalência de dentição funcional entre pessoas brancas, pretas e pardas, reforçando a permanência da estrutura de iniquidade sob o desfecho. No tocante à avaliação da PNSB e das políticas mitigadoras de iniquidades, isso aponta para um aspecto importante nas suas ações e serviços: é possível que estejam centradas na redução dos agravos propriamente ditos, deixando em segundo plano a redução das iniquidades.

Esse resultado é consistente com evidências anteriores que demonstram piores indicadores de saúde bucal entre pessoas negras, incluindo maior prevalência de edentulismo, maior ocorrência de cárie não tratada e menor acesso a procedimentos restauradores, mesmo após considerar diferenças socioeconômicas^37,38^. A persistência dessas desigualdades sugere que as diferenças observadas não podem ser compreendidas apenas pelas características individuais, mas estão relacionadas a processos históricos e estruturais.

Dados da Pesquisa Nacional de Saúde (PNS) de 2019 reforçam essa interpretação ao demonstrarem que adultos negros e pardos apresentam maior probabilidade de não terem realizado consulta odontológica no último ano, mesmo após ajustes por região e renda^23^. Esses achados indicam que a cor da pele ou raça permanece associada a desigualdades no acesso e na utilização dos serviços odontológicos, refletindo mecanismos relacionados à discriminação institucional e à distribuição desigual de recursos e oportunidades de cuidado. Padrão semelhante tem sido descrito em diferentes contextos internacionais, incluindo Estados Unidos^39^, Reino Unido^40^ e Canadá^41^, indicando que desigualdades raciais em saúde podem persistir mesmo em sistemas de saúde estruturados.

Entre os pontos fortes deste estudo destacam-se a utilização de inquéritos nacionais de saúde bucal realizados em diferentes períodos históricos e a aplicação de uma abordagem analítica capaz de investigar como idade e período se relacionam na evolução da dentição funcional. A análise de cortes transversais repetidos permitiu avaliar mudanças populacionais ao longo de duas décadas e identificar que os ganhos observados não ocorreram de maneira uniforme entre os grupos etários. Além disso, a incorporação da cor da pele ou raça possibilitou analisar a persistência de desigualdades sociais em um desfecho relevante para a saúde bucal e para o envelhecimento saudável.

Algumas limitações devem ser consideradas, por se tratar de dados provenientes de inquéritos transversais repetidos. Não foi possível acompanhar indivíduos ao longo do tempo nem distinguir completamente os efeitos relacionados à coorte, apenas a idade e o período. Mesmo que a interação entre idade e período permita investigar diferenças entre grupos etários em distintos momentos históricos, seus resultados devem ser interpretados considerando as limitações inerentes aos desenhos observacionais. Além disso, a mensuração da dentição funcional baseada na presença de 20 ou mais dentes remanescentes^3^, amplamente utilizada em estudos epidemiológicos, não incorpora aspectos clínicos e funcionais específicos, como a presença de pares oclusais, distribuição dos dentes remanescentes, necessidade de tratamento ou impacto funcional da condição dentária.

O conjunto de achados sugere que a saúde bucal brasileira atravessa uma mudança de paradigma. As novas gerações tendem a envelhecer mantendo um número cada vez maior de dentes naturais, impondo novos desafios aos serviços odontológicos, que passam a atender uma população idosa com necessidades distintas das observadas em gerações anteriores. Essa transição, no entanto, ocorre de forma desigual; enquanto os ganhos entre gerações podem revelar o impacto de melhorias sociais e assistenciais acumuladas nas últimas décadas, a persistência das diferenças raciais evidencia que o progresso populacional, por si, não é suficiente para reduzir iniquidades estruturais. O citado sugere que políticas orientadas predominantemente para a melhoria dos indicadores médios de saúde podem produzir importantes avanços, sem se voltar à redução de desigualdades estruturais. O desafio passa a ser não apenas ampliar a longevidade dentária da população, mas garantir que a atuação da política seja uma *proxy* da mitigação de iniquidades.

## Material Suplementar

Material Suplementar
